# Phosphorylation State-Dependent Interactions of Hepadnavirus Core Protein with Host Factors

**DOI:** 10.1371/journal.pone.0029566

**Published:** 2011-12-22

**Authors:** Laurie Ludgate, Christina Adams, Jianming Hu

**Affiliations:** Department of Microbiology and Immunology, The Pennsylvania State University College of Medicine, Hershey, Pennsylvania, United States of America; Yonsei University, Republic of Korea

## Abstract

Dynamic phosphorylation and dephosphorylation of the hepadnavirus core protein C-terminal domain (CTD) are required for multiple steps of the viral life cycle. It remains unknown how the CTD phosphorylation state may modulate core protein functions but phosphorylation state-dependent viral or host interactions may play a role. In an attempt to identify host factors that may interact differentially with the core protein depending on its CTD phosphorylation state, pulldown assays were performed using the CTD of the duck hepatitis B virus (DHBV) and human hepatitis B virus (HBV) core protein, either with wild type (WT) sequences or with alanine or aspartic acid substitutions at the phosphorylation sites. Two host proteins, B23 and I2PP2A, were found to interact preferentially with the alanine-substituted CTD. Furthermore, the WT CTD became competent to interact with the host proteins upon dephosphorylation. Intriguingly, the binding site on the DHBV CTD for both B23 and I2PP2A was mapped to a region upstream of the phosphorylation sites even though B23 or I2PP2A binding to this site was clearly modulated by the phosphorylation state of the downstream and non-overlapping sequences. Together, these results demonstrate a novel mode of phosphorylation-regulated protein-protein interaction and provide new insights into virus-host interactions.

## Introduction

Hepadnaviruses, or hepatitis B viruses, are hepatotropic DNA viruses, consisting of an enveloped icosahedral capsid containing an approximately 3 kb DNA genome in a partially double-stranded, relaxed circular form (RC DNA). The virus carries its own polymerase enzyme, a reverse transcriptase, that converts the packaged pregenomic RNA (pgRNA) to RC DNA through reverse transcription inside the capsid [1]. The capsid is formed by multiple copies (180 or 240) of one protein, the capsid or core protein [2]–[5]. The core protein is composed of two separate domains: the N-terminal domain (NTD) that is sufficient to form the capsid shell and the C-terminal domain (CTD) that is dispensable for capsid assembly but nevertheless essential for viral replication [6]–[8].

The CTD is highly basic, rich in arginines, but also contains multiple sites of serine/threonine (S/T) phosphorylation [8]–[11], which, when phosphorylated, partially neutralize the positive charges of the CTD and also induce conformational changes [9]. The human hepatitis B virus (HBV) core protein (HBc) contains three major S phosphorylation sites at its CTD [10]. Similarly, duck hepatitis B virus (DHBV) core protein (DHBc) contains six phosphorylation sites at its CTD [9], [11]. Phosphorylation of hepadnavirus core protein has been shown to play multiple roles in viral replication. HBc CTD phosphorylation is important for RNA packaging and DNA synthesis [7], [12], [13]. DHBc CTD phosphorylation appears to play only a minor role in RNA packaging but is essential for the early stage of viral DNA synthesis [8], [14]–[16]. Moreover, the phosphorylated DHBc CTD becomes completely dephosphorylated during the late stage of viral DNA synthesis [11], which is required for viral DNA maturation and stability [15]. These results have led to a model of dynamic DHBc CTD phosphorylation whereby CTD phosphorylation is required for minus-strand DNA synthesis and dephosphorylation required for the synthesis/accumulation of mature double-stranded DNA.

For both HBV and DHBV, the core protein has been detected in both the cytoplasm and the nucleus [10], [17]–[20]. While capsid assembly and DNA synthesis are known to take place in the cytoplasm, the function for core protein in the nucleus, if any, remains unresolved. It has been suggested that the core protein escorts the RC DNA genome into the nucleus, where it is converted to the covalently-closed circular (CCC) DNA form that serves as the template for viral transcription and maintains persistent infections [21], [22]. Core protein phosphorylation has been reported to affect its nuclear localization and thus potentially RC DNA nuclear import [10], [18], [20], [23].

It is still unclear how the state of phosphorylation regulates core protein function in RNA packaging, DNA synthesis, nuclear import, and potentially additional steps in the viral life cycle. One possibility is that the core protein exerts its multiple roles by interacting dynamically with distinct viral or host factors at different stages of viral replication, in a CTD phosphorylation state-dependent fashion. We have now indeed identified cellular proteins that interacted preferentially with the unphosphorylated CTD and could potentially modulate viral replication.

## Results

### DHBc CTD phosphorylation mutants exhibited replication phenotypes in HEK293T cells similar to those in LMH cells

**Figure 1:**
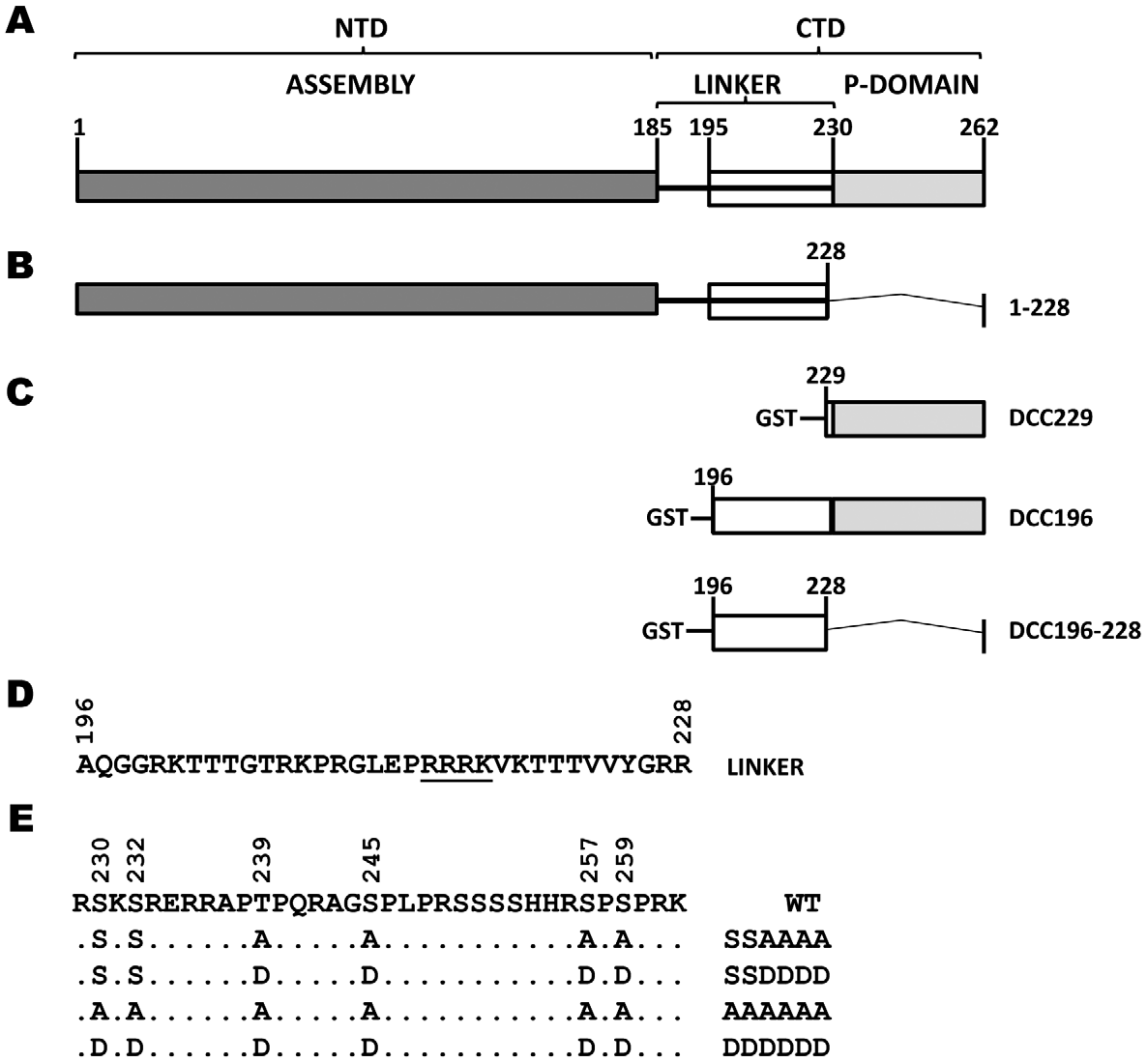
Schematic diagram of DHBc domains and GST-DHBc CTD (DCC) fusion constructs. *A.* DHBc contains an N-terminal assembly domain (NTD) from amino acids 1–185, a flexible linker region from amino acid 186 that extends into the CTD, which includes the phosphodomain from 230–262. *B.* 1–228, a CTD-deletion mutant. *C.* Each WT fusion protein contains a differing length of the CTD and was named for the amino acid position where the tail fragment begins (229 or 196). DCC229 contains the phosphodomain from residue 229 with all six known WT phosphorylation sites (S230, S232, T239, S245, S257, and S259, *panel E*). DCC196 contains the phosphodomain plus the additional upstream 33 amino acids of the linker region. DCC196-228 contains amino acid residues 196–228 (the linker region), deleting the entire phosphodomain with all six phosphorylation sites. *D.* The linker sequence with the basic cluster underlined. *E.* Alanine (A) or aspartic acid (D) substitutions were made in the full-length DHBc, as well as in the context of each WT fusion protein, with the resultant mutants the same length as the WT version. The full-length A and D mutants were named by the positions of the mutated phosphosites they contain: SSAAAA, SSDDDD, AAAAAA, and DDDDDD. The GST-DCC fusion proteins were named by their amino acid starting position and their phosphosite substitutions. The final four phosphorylation sites were substituted by A in 229-SSAAAA or D in 229-SSDDDD. 196-SSAAAA contained amino acids 196–262 with the last four phosphorylation sites mutated to A and maintained S230 and S232. Similarly, 196-SSDDDD maintained S230 and S232, while the last four phosphosites contained D substitutions. Finally, all six phosphorylation sites were substituted by A in 196-AAAAAA or D in 196-DDDDDD.

**Figure 2:**
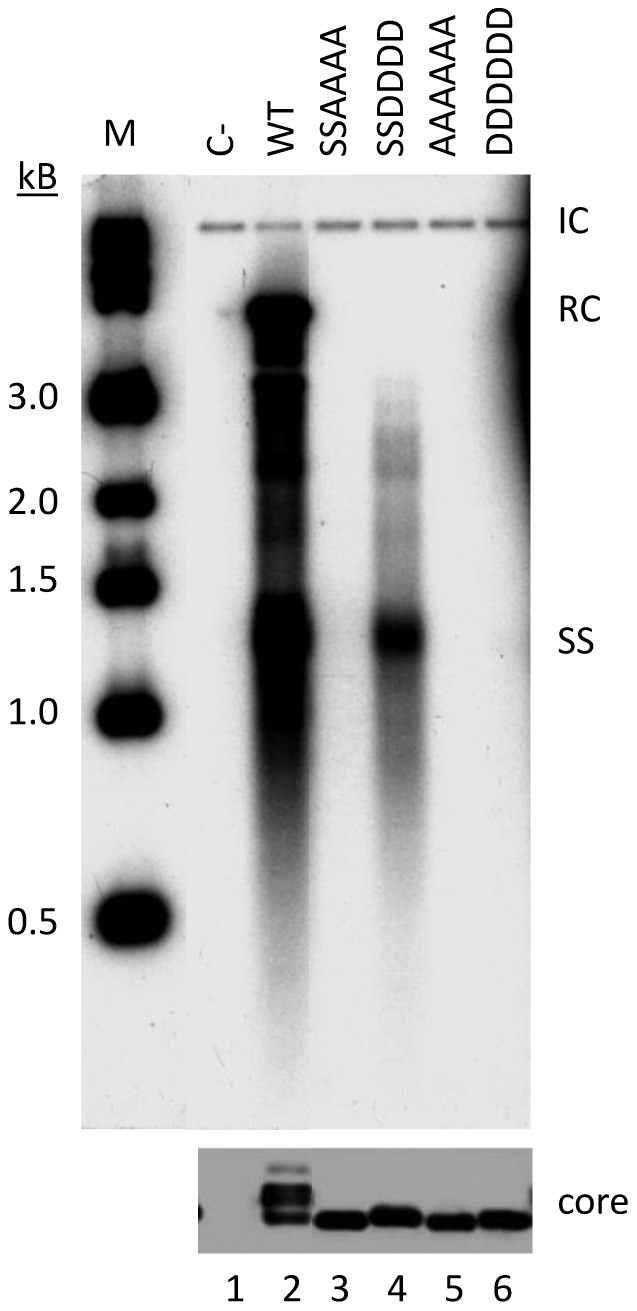
Effect of DHBc phosphorylation site mutations on protein expression and DNA synthesis in HEK293T cells. HEK293T cells were cotransfected with pCMV-DHBV/C-, which expresses a core-defective DHBV genome, and the indicated WT or mutant core expression plasmid. Five days post-transfection, cells were lysed and core DNA was isolated as previously described. DHBc was detected by western blotting with antibody against DHBc (*bottom panel*) and core DNA was detected by Southern blotting analysis with a DHBV DNA probe (*top panel*). RC, relaxed-circular DNA; SS, single-stranded DNAs; IC, internal control plasmid added during the DHBV DNA extraction.

We previously reported that during DHBV reverse transcription, phosphorylation of the DHBc CTD is required for first strand DNA synthesis while its dephosphorylation is required for second strand maturation [15]. These findings were originally described using LMH cells and we were interested in determining if this requirement was cell-type or species specific. To this end, we transfected the human embryonic kidney cell line, HEK293T, which is known to support DHBV and HBV replication [24], [25], with the DHBc mutants containing substitutions that either block (S/T to A) or mimic (S/T to D) phosphorylation at the CTD phosphorylation sites (**Fig. 1**) and analyzed the effect of these substitutions on DHBc expression and viral DNA replication (**Fig. 2**). We observed a similar phenotype in HEK293T cells to what we reported in LMH cells [15]. Mutants that blocked phosphorylation of the CTD (S/T to A) at the four downstream or all six phosphorylation sites were unable to synthesize any DNA (**Fig. 2**, lanes 3 and 5). The SSDDDD mutant was blocked from accumulating RC DNA, showing only single-stranded intermediate species (**Fig. 2**, lane 4), and the DDDDDD mutant accumulated only barely detectable single-stranded DNA (**Fig. 2**, lane 6). Also, DHBc expression levels in HEK293T cells, as in LMH cells, were not affected by the phosphodomain substitutions (**Fig. 2**, bottom), although, as in LMH cells, the phosphorylation-dependent migrational heterogeneity on sodium dodecyl sulfate polyacrylamide gel electrophoresis (SDS-PAGE) was abolished by the A or D substitutions at the S/T-proline (P) motifs (**Fig. 1E**; **Fig. 2**, lanes 3–6) [9], [15].

### Identification of cellular proteins interacting with the DHBc CTD in a phosphorylation state-dependent manner

**Figure 3:**
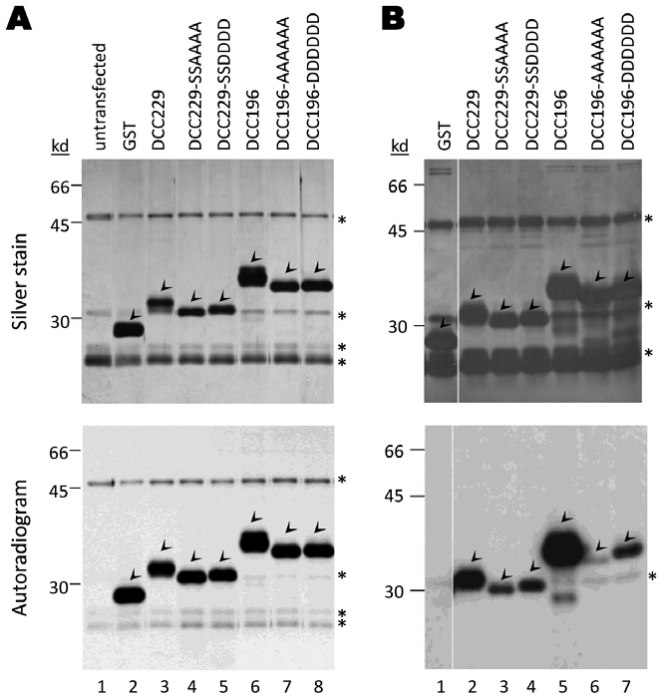
Metabolic labeling of GST-DCC constructs in HEK293T cells. Cells were transfected with the indicated plasmids expressing either GST or GST-DCC fusion proteins, then were metabolically labeled with either [^35^S]cysteine/methionine or [^32^P]orthophosphate on the third day post-transfection. *A.* ^35^S- or *B.* ^32^P-labeled proteins were purified without RNase A digestion using GSH affinity resin and were visualized by silver staining (*top panels*) and autoradiography (*bottom panels*). Arrowheads indicate GST or GST-DCC fusion proteins. *, non-specific background bands that appeared in all transfections or untransfected cells.

One mechanism by which the core protein phosphorylation state regulates core protein functions may be via phosphorylation state-dependent interactions with viral or host factors. To test for the interactions between DHBc and potential host factors, particularly those interactions at the CTD that are modulated by the state of CTD phosphorylation, we constructed GST fusion proteins containing the DHBc CTD (GST-DCC), with WT sequences or phosphorylation sites substituted with A or D (**Fig. 1E**). We first tested these GST-fusion proteins in both LMH and HEK293T cell lines to verify that the fusion proteins were phosphorylated (or prevented from phosphorylation) as anticipated. [^35^S]cysteine/methionine and [^32^P]orthophosphate metabolic labeling showed that the proteins were expressed in both cell lines and that they were indeed phosphorylated as expected (**Fig. 3B**). As observed with the full-length DHBc, the WT CTD, with or without the so-called linker region between the NTD and the phosphodomain (**Fig. 1**), exhibited the characteristic mobility heterogeneity in both LMH (data not shown) and HEK293T cells (**Fig. 3**), which was abolished by the A or D substitutions at the four S/T-P phosphorylation sites, just as in the case of the full-length proteins [15]. These results indicated that the isolated CTD, when fused to GST, behaved similarly to that in the full-length DHBc both in terms of phosphorylation and phosphorylation-induced conformational changes as manifested by the migrational heterogeneity [9]. Because the fusion proteins were expressed much more efficiently in HEK293T cells than in LMH cells (data not shown) due to the high transfection efficiency of the former, and the DHBc phosphomutants had similar phenotypes in HEK293T and LMH cells, most of the binding studies were carried out in HEK293T cells.

**Figure 4:**
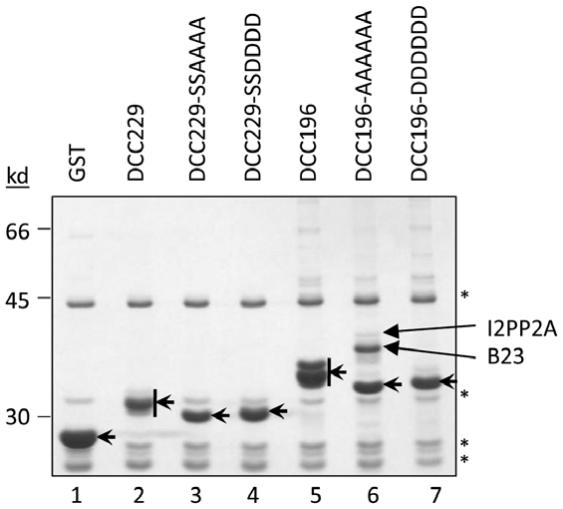
GST co-purification and MS analysis. HEK293T cells were transfected with the indicated GST-DCC fusion constructs. GST fusion proteins (short arrows) were purified from RNase A-treated lysates using GSH affinity resin. Co-purifying proteins were resolved by SDS-PAGE and detected by CB staining. Protein bands of interest were excised, subjected to in-gel trypsinization, and identified by mass spectrometry (long arrows). *, non-specific background bands that appeared in all samples, including GST alone.

To test for host factor interaction, we transfected HEK293T cells with the GST-DCC fusion protein expression constructs and performed GST pulldown experiments. We observed no detectable co-purifying bands in any of the fusion proteins containing only the phosphodomain, either as WT or phosphomutants (229, 229-SSAAAA and 229-SSDDDD) (**Fig. 4**, lanes 2–4). Only when we extended the fusion proteins to include the upstream linker region starting at residue 196 (**Fig. 1**) were we able to detect host factor interactions. We noted that detection of host factor interaction was facilitated by treating the cell lysates with RNase A (see Materials and Methods), as shown in **Fig. 4**. In contrast, the experiments shown in **Fig. 3** did not reveal consistent co-purifying host proteins above background where RNase A treatment was omitted. Especially striking were the strong ca. 38 kD band in the 196-AAAAAA sample (**Fig. 4**, lane 6) and the less intense band migrating just above it at ca. 40 kD in the same sample. These bands were not apparent in any other samples, suggesting that these putative host factors interacted preferentially with the unphosphorylated CTD. In-gel trypsin digestion followed by mass spectrometry identified the 38 kD band as B23 and the 40 kD band as I2PP2A.

**Figure 5:**
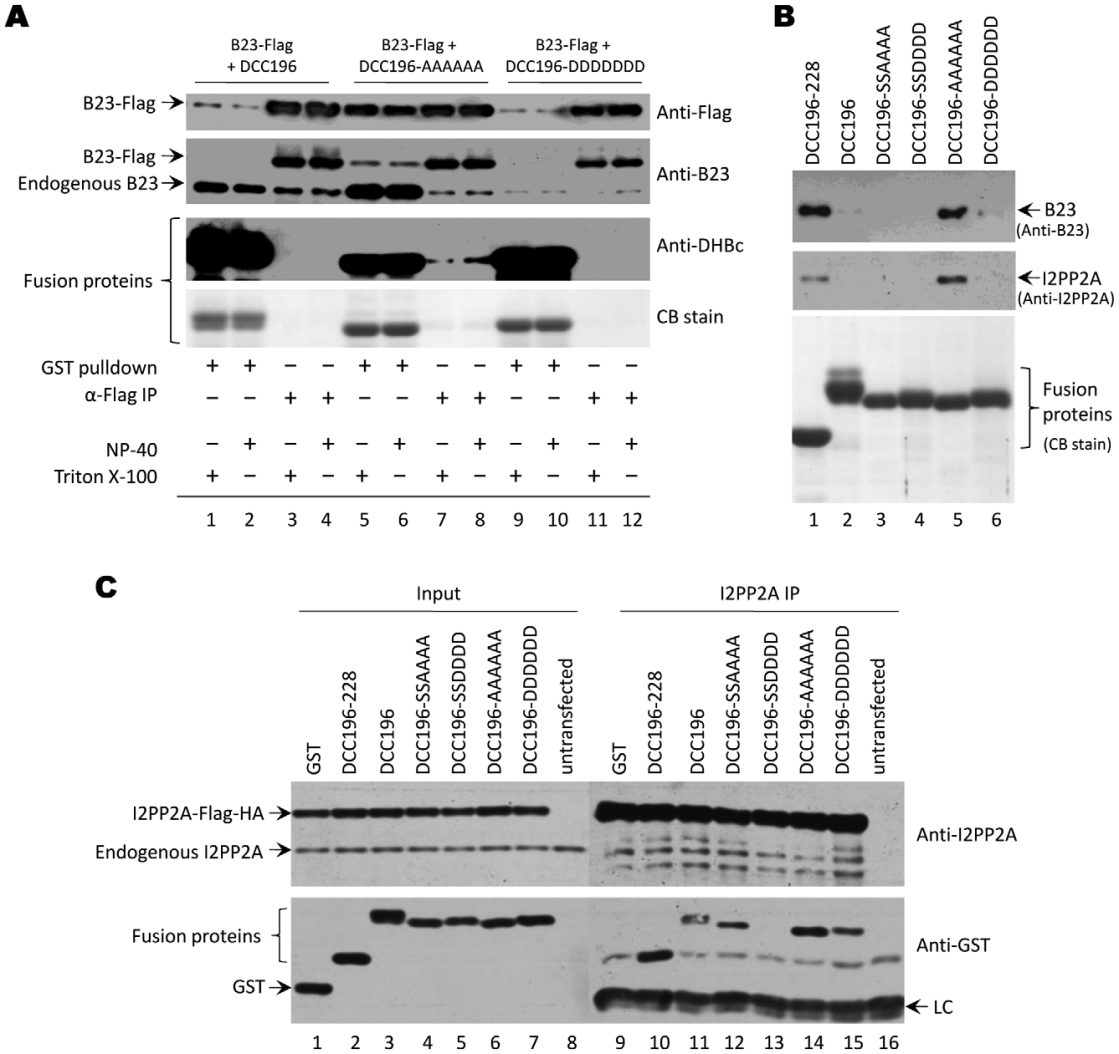
Coimmunoprecipitation and pulldown experiments. *A.* HEK293T cells were cotransfected with plasmids to express GST-DCC fusion constructs and 3X-Flag-tagged B23 protein. Coimmunoprecipitation experiments were conducted with an anti-Flag antibody. GST pulldown, using GSH affinity resin, was performed in parallel. *B.* HEK293T cells were transfected with GST-DCC fusion protein expression plasmids. GST pulldown, using GSH affinity resin, was performed. Samples were analyzed by western blotting with antibodies to detect B23 (*top panel*) and I2PP2A (*middle panel*). *C.* HEK293T cells were cotransfected with plasmids to express GST-DCC fusion proteins and I2PP2A-Flag-HA. Immunoprecipitation was conducted with a mixture of antibodies against Flag and HA. Samples were analyzed by western blotting with antibodies to detect I2PP2A (*top panel*) and GST (*bottom panel*). LC, light chain.

To confirm the interaction between GST-DCC fusion proteins and B23 or I2PP2A, we performed coimmunoprecipitation experiments using 3X-Flag-tagged B23 or Flag- and HA- double-tagged I2PP2A. Additionally, since the 229 constructs, in contrast to the 196 constructs, did not show any host factor interaction, we constructed another fusion protein, containing only the linker region from 196–228 (**Fig. 1D**), in order to test if this segment of the CTD by itself might interact with B23 or I2PP2A. As anticipated, 196-AAAAAA coimmunoprecipitated with B23 (**Fig. 5A**, lanes 7 and 8) but neither did the WT 196 fusion protein (**Fig. 5A**, lanes 3 and 4) nor the 196-DDDDDD mutant (**Fig. 5A**, lanes 11 and 12). In the GST co-purification experiment conducted in parallel, B23 interaction was again strongest with 196-AAAAAA (**Fig. 5A**, lanes 5 and 6; also see **5B**, lane 5). Barely detectable B23 interaction could be seen in GST co-purifications performed with 196 WT and 196-DDDDDD (**Fig. 5A**, lanes 1 and 2, 9 and 10; also see **5B**, lanes 2 and 6). The use of NP-40 or Triton X-100 in the lysis buffer did not make any difference in these interactions.

As with B23, the 196-AAAAAA interaction with I2PP2A was stronger than 196-DDDDDD (**Fig. 5C** **,** lane 14 vs. 15). I2PP2A interaction could also be detected with 196-SSAAAA (**Fig. 5C**, lane 12), although it was weaker than 196-AAAAAA, and no interaction was detected between I2PP2A and 196-SSDDDD (**Fig. 5C**, lane 13). Interestingly, the linker region, 196–228, showed an interaction with B23 and I2PP2A as strong as 196-AAAAAA (**Fig. 5B**, lanes 1 and 5; **Fig. 5C**, lanes 10 and 14). These results combined indicated that B23 and I2PP2A were actually binding to the linker region (196–228) but this interaction was modulated by the phosphorylation state of the downstream phosphodomain.

**Figure 6:**
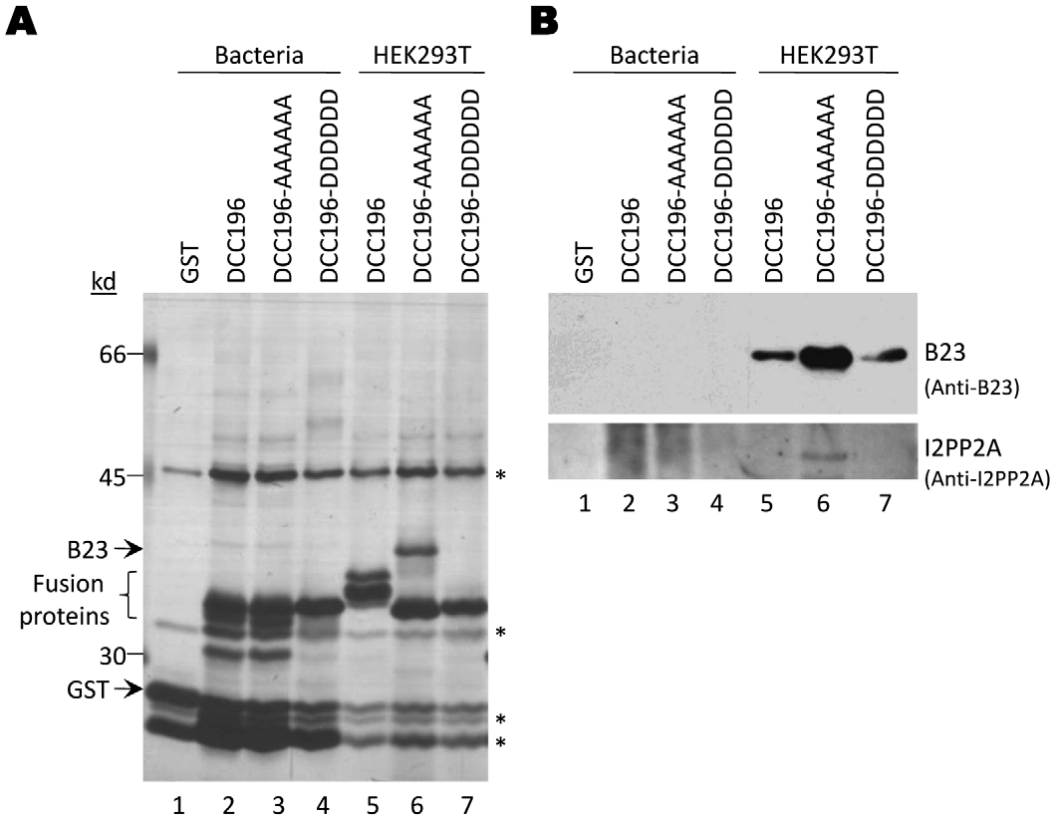
GST pulldown with *E. coli*-derived DCC fusion proteins. GST or GST-DCC fusion proteins were purified from *E. coli*. The purified proteins were added to RNase A-treated HEK293T cell lysates. HEK293T cellular proteins that were pulled down by the fusion proteins were visualized by silver staining (*A*, lanes 1–4) and western blotting (*B*, lanes 1–4). For comparison, the same GST-DCC fusion proteins were expressed and purified from HEK293T cells and the co-purifying cellular proteins were shown in *A & B*, lanes 5–7. *, non-specific background bands that appeared in all samples, including GST alone.

In contrast to the GST-DCC fusion proteins expressed in HEK293T cells, none of the bacterially-expressed fusion proteins showed any binding to B23 or I2PP2A from HEK293T cell lysate (**Fig. 6A and B**, lanes 1–4), suggesting that the *E. coli*-derived proteins may be folded differently from those expressed in mammalian cells and therefore could not bind B23 or I2PP2A. An alternative interpretation that co-translational binding was necessary was ruled out by the experiment shown below using an in vitro translation system.

### Full-length DHBV core protein interacted with B23 and I2PP2A

**Figure 7:**
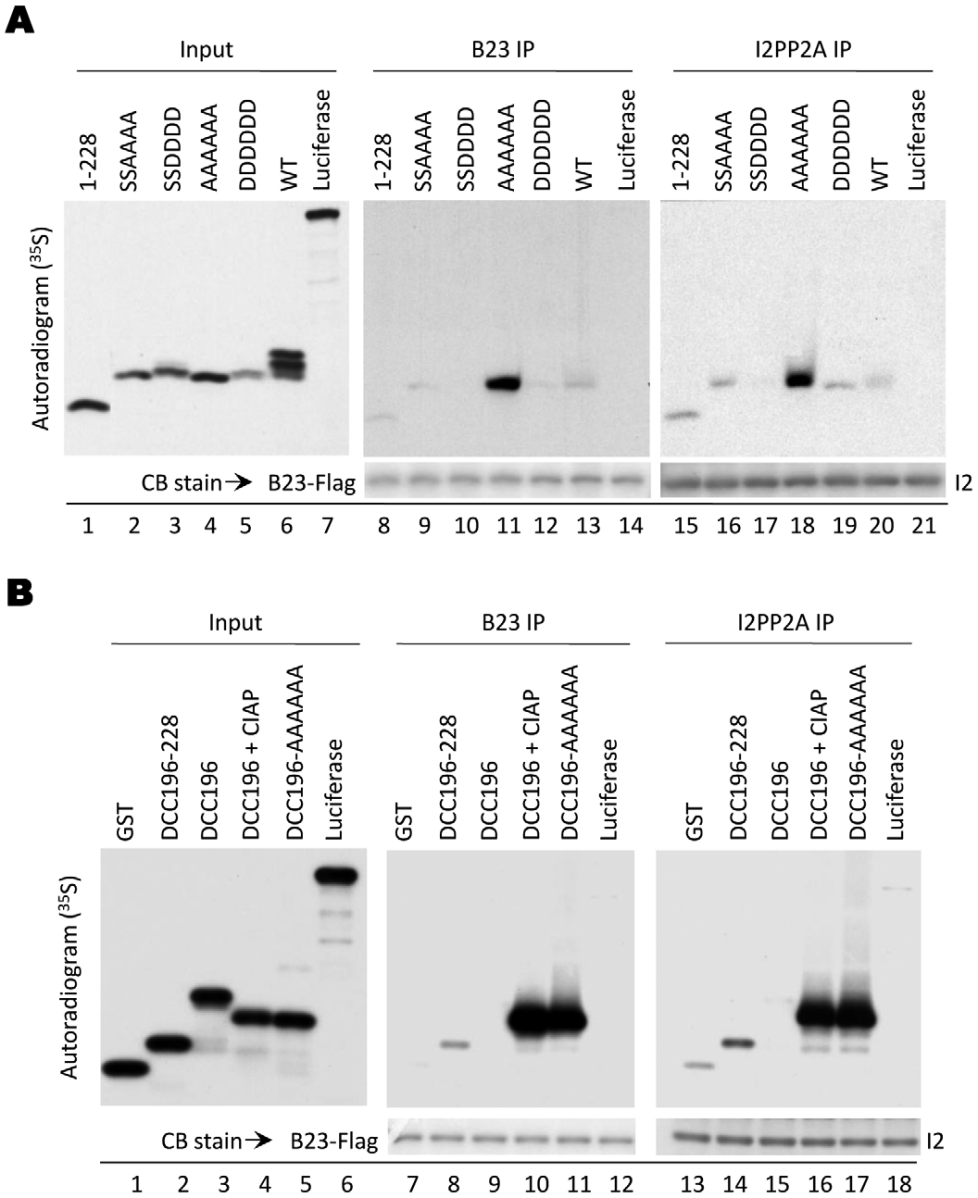
Interaction between the in vitro-translated full-length DHBc proteins or GST-DCC fusion proteins and B23 and I2PP2A. *A.* Full-length DHBc proteins or firefly luciferase were translated in RRL and interaction with B23 or I2PP2A purified from HEK293T cells was determined by coimmunoprecipitation. Lanes 1–7 show RRL-translated input proteins (autoradiogram). Lanes 8–14 show the B23 immunoprecipitate (autoradiogram, *top panel*, and CB stain, *bottom panel*) and lanes 15-21 show the I2PP2A immunoprecipitate (autoradiogram, *top panel*, and CB stain, *bottom panel*). *B.* GST-DCC fusion proteins were translated in RRL and interaction with B23 or I2PP2A purified from HEK293T cells was determined by coimmunoprecipitation. The WT DCC196 fusion protein was either dephosphorylated with CIAP or left untreated. Lanes 1–6 show RRL-translated input proteins. Lanes 7–12 show the B23 immunoprecipitate (autoradiogram, *top panel*, and CB stain, *bottom panel*) and lanes 13–18 show the I2PP2A immunoprecipitate (autoradiogram, *top panel*, and CB stain, *bottom panel*). I2, I2PP2A-Flag-HA.

To address whether full-length DHBc would interact with either B23 or I2PP2A, we expressed the WT and mutant core proteins in the rabbit reticulocyte lysate (RRL) translation system and asked if B23 or I2PP2A purified from HEK293T cells could coimmunoprecipitate the in vitro-translated proteins. Attempts to detect interactions between full-length core protein expressed in HEK293T cells and B23 or I2PP2A were unsuccessful, likely due to the relatively low expression levels (compared to the fusion proteins) and the rapid assembly of full-length core proteins to capsids in cells. The in vitro-translated full-length DHBc containing the AAAAAA substitutions indeed interacted with both B23 and I2PP2A strongly (**Fig. 7A**, lanes 11 and 18), as was observed with the GST-fusion constructs. Full-length DDDDDD interaction with B23 or I2PP2A was much weaker than AAAAAA (**Fig. 7A**, lanes 12 and 19), and the SSAAAA interactions (**Fig. 7A**, lanes 9 and 16) were much weaker than AAAAAA but better than SSDDDD, which did not show detectable binding to either B23 or I2PP2A (**Fig. 7A**, lanes 10 and 17). Most interestingly, the fastest-migrating band of the WT full-length DHBc, representing the hypo- or non-phosphorylated form, but not the hyperphosphorylated, slower-migrating species, also bound to both B23 and to I2PP2A (**Fig. 7A**, lanes 13 and 20).

To compare the CTD fusion protein and full-length DHBc interactions with B23/I2PP2A in the same system, under the same conditions, we also expressed the GST-DCC fusion proteins in the RRL system. Again, we saw very strong, specific interaction between both B23 and I2PP2A and 196-AAAAAA (**Fig. 7B**, lanes 11 and 17). Compared to the WT full-length DHBc, the WT 196 fusion protein was even more heavily phosphorylated in RRL, as indicated by the single intense, slower-migrating band, and did not interact with either B23 or I2PP2A. Because the hypo-phosphorylated (or non-phosphorylated) full-length core protein interacted with B23 and I2PP2A, we attempted to dephosphorylate the WT CTD and see if the dephosphorylated CTD would gain the ability to interact with B23 and I2PP2A. Thus, we treated the WT CTD with calf intestinal alkaline phosphatase (CIAP) and indeed observed a dramatic downward shift so that it now co-migrated with 196-AAAAAA (**Fig. 7B**, lane 4), indicating rather complete dephosphorylation of the WT CTD. Strikingly, we now saw that the dephosphorylated WT CTD strongly interacted with both B23 and I2PP2A to a level similar to the 196-AAAAAA mutant (**Fig. 7B**, lanes 10 and 16). These results confirmed the importance of the unphosphorylated state of the CTD in allowing the specific interactions of the core protein with B23 and I2PP2A.

We observed only a weak interaction of the linker region construct (196–228), expressed in RRL, with either B23 or I2PP2A (**Fig. 7B**, lanes 8 and 14), in contrast to the strong binding (as strong as that of 196-AAAAAA) of this same region when it was expressed in HEK293T cells. The corresponding full-length construct (1–228) containing the linker but not the phosphodomain, when expressed in RRL, also interacted only weakly with B23 and I2PP2A (**Fig. 7A**, lanes 8 and 15), suggesting that the interactions between the core protein linker region, in the absence of the phosphodomain, and B23/I2PP2A were influenced by the system used to express the linker sequences.

### The CTD of HBc interacted with B23 and I2PP2A in a phosphorylation state-dependent manner

**Figure 8:**
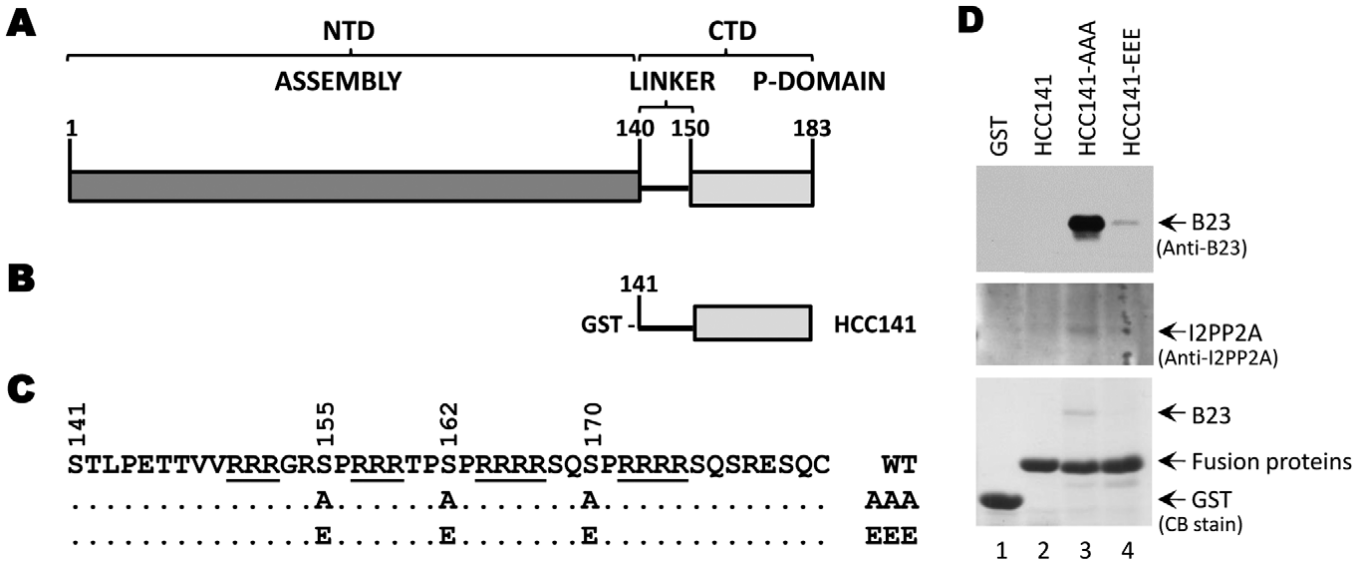
Interaction of the HBc CTD with B23 and I2PP2A. *A.* Schematic diagram of HBc domains. Depicted are the N-terminal assembly domain (NTD) from amino acids 1–140 and the CTD that includes a flexible linker region from amino acid 141–149 and a phosphodomain from 150–183. *B.* GST-HBc CTD (HCC) fusion construct. The WT HCC141 fusion protein contains the CTD from residue 141 to the end of HBc. *C.* The sequence of the HBc CTD, with the three major phosphorylation sites (S155, S162, and S170) and alanine (A) or glutamic acid (E) substitutions indicated. The phosphosite mutant GST-HCC fusion proteins were named for the mutated phosphosites they contain: HCC141-AAA or HCC141-EEE. The four basic clusters are underlined. *D.* CB staining (*bottom panel*) and western blotting (*top* and *middle panels*) analysis of GST (lane 1) and GST-DCC fusion proteins (lanes 2–4) purified from HEK293T cells.

We next asked whether this phosphorylation state-dependent interaction would also apply to HBc. We thus constructed GST-fusion proteins containing the CTD of HBc either with WT sequences or with the three major phosphorylation sites substituted with alanine or glutamic acid (**Fig. 8B and C**), similar to the approach taken with DHBc. We expressed the GST-HBc CTD (HCC) fusion constructs in HEK293T cells and performed pulldown experiments.

Consistent with the results that we obtained with DHBc, we found that the HCC fusion protein containing A substitutions (HCC141-AAA) and representing the hypo- or non-phosphorylated CTD showed strong interaction with B23 (**Fig. 8D**, lane 3, *top & bottom panel*). Interaction was also seen between the HCC141-AAA mutant and I2PP2A (**Fig. 8D**, lane 3, *middle panel*). Also similar to DHBc observations, interaction between HCC141-EEE, representing the phosphorylated CTD, and B23 was much weaker (**Fig. 8D**, lane 4, *top panel*) and HCC141-EEE and I2PP2A interaction was undetectable (**Fig. 8D**, lane 4, *middle panel*). No interaction was detectable between the WT HBc CTD and either B23 or I2PP2A in these experiments (**Fig. 8D**, lane 2).

## Discussion

In this report, we have demonstrated the preferential interaction of unphosphorylated hepadnavirus core protein with two host factors: B23 (also known as nucleophosmin and NPM1) and I2PP2A (also known as SET and Taf-1β). These interactions were identified by using GST-fusion proteins containing the isolated CTDs of DHBc and HBc and were confirmed for the full-length DHBc. Although the DHBc binding site for both host factors was localized to the linker region (196–228) upstream of the CTD phosphodomain, for both the isolated CTD and the full-length core protein, host factor interactions were nevertheless strongly modulated by the phosphorylation state of the downstream CTD phosphodomain.

**Figure 9:**
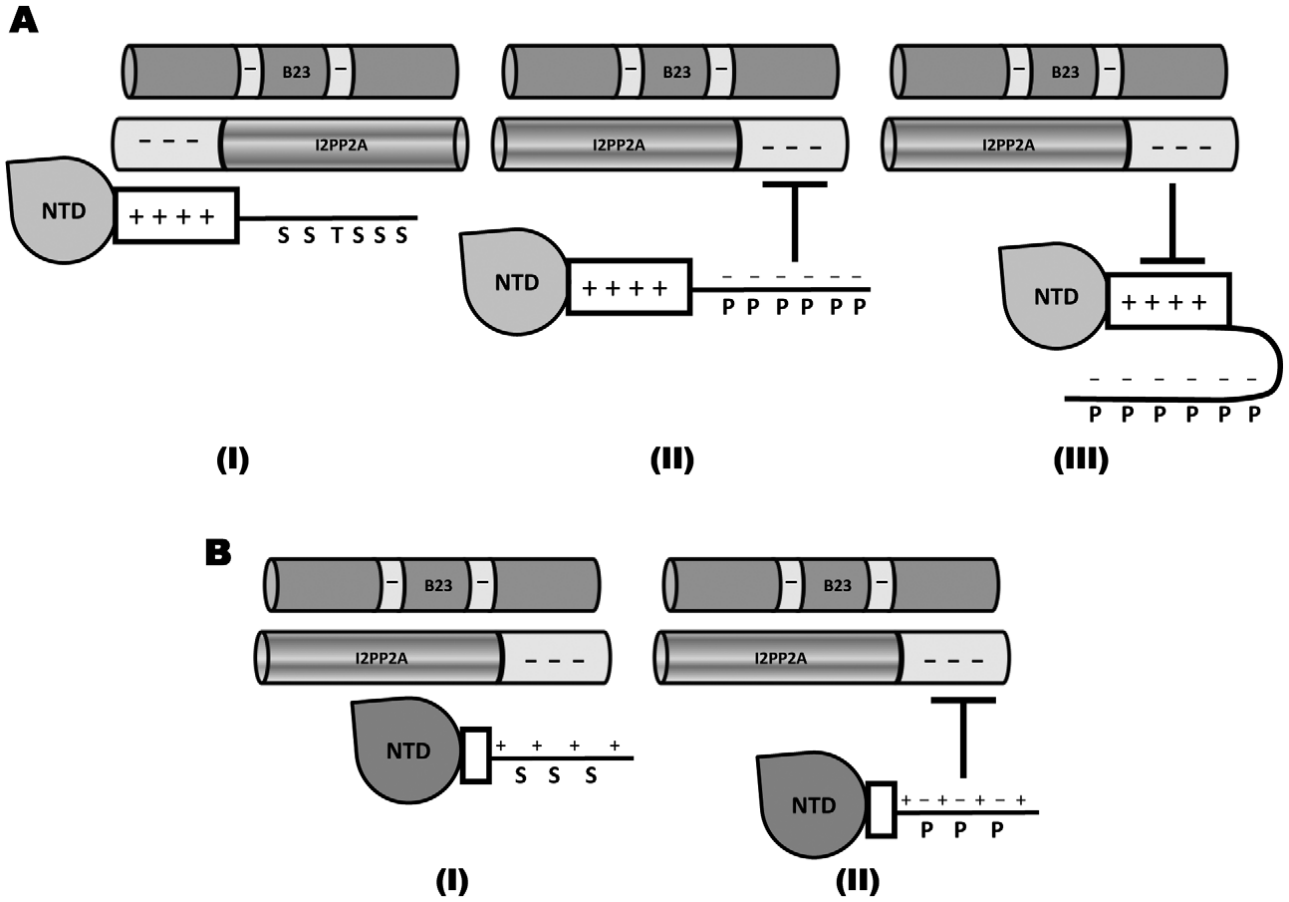
Possible mechanisms for the differential binding of phosphorylated and unphosphorylated hepadnavirus core protein to B23 and I2PP2A. *A.* The lack of phosphorylation in the DHBc phosphodomain permits an open conformation in the CTD and renders the upstream linker region containing the host protein binding site accessible for B23/I2PP2A interactions (*I*). The negative charges introduced by CTD phosphorylation decrease the overall positive charge of the CTD and thus weaken the electrostatic interactions with the acidic regions of B23 and I2PP2A (*II*). Alternatively, the negative charges introduced into the phosphodomain may enable it to fold over and interact with the basic upstream linker and thereby mask the host protein binding site located within the linker (*III*). *B.* Similarly, phosphorylation of the HBc CTD would result in a decreased overall positive charge of the CTD that weakens the electrostatic interactions with the acidic regions of B23 and I2PP2A (*I* and *II*). In this case, the positively charged host protein binding sites are interspersed with the phosphorylation sites.

There have been a few reports on cellular factors that interact with HBc, including a 46-kD serine kinase [26], SRPK1 [27], TAP/NXF1 [28], β-importin [21], heat shock protein (hsp) 40 [29], and hsp 90 [30]. Among these, the binding of β-importin appears to require HBc phosphorylation [21]. B23 and I2PP2A represent the first host proteins shown to interact preferentially with a non-phosphorylated hepadnavirus core protein (HBc and DHBc) over the phosphorylated species. Most interestingly, these host proteins did not interact directly with the DHBc phosphodomain but instead bound further upstream to the so-called “linker region” between the NTD and the phosphodomain. How the phosphorylation state of the DHBc CTD phosphodomain influences B23 or I2PP2A binding to the linker region remains to be elucidated. The strong positive charge of the linker region and the presence of acidic domains in B23 and I2PP2A suggest that electrostatic interactions may contribute significantly to the CTD-B23 or -I2PP2A interactions (**Fig. 9A**, *panel I*), and CTD phosphorylation, by partially neutralizing the overall positive charge of the CTD, could weaken the host interactions (**Fig. 9A**, *panel II*). It is also possible that the phosphorylated CTD could fold back to interact with the positively charged linker and thus preclude the linker-host interactions, if negatively charged patches are formed in the phosphodomain as a result of phosphorylation (**Fig. 9A**, *panel III*). HBc CTD-B23 and -I2PP2A interactions were similarly phosphorylation state-dependent. The HBc linker region is much shorter (9 amino acids) (**Fig. 8**) and does not contain any basic residues. Instead, the HBc phosphodomain itself contains four clusters of arginines and presumably harbors the B23 and I2PP2A binding site(s) (**Fig. 8C** and **9B**, *panel I*). Introduction of negative charges next to the basic clusters by phosphorylation of the neighboring serine residues would decrease their positive charges and weaken the interactions with B23 and I2PP2A (**Fig. 9B**, *panel II*). However, as discussed below, electrostatic interaction alone is unlikely to mediate the observed CTD-B23 or I2PP2A interaction.

GST-DCC fusion proteins that were purified from *E. coli* did not exhibit any detectable interactions with eukaryotic proteins. The fusion protein with WT sequences was not phosphorylated in bacteria, so it might have been predicted that the WT fusion protein made in *E. coli* would behave similarly to 196-AAAAAA. The lack of any interaction by the bacterially-expressed fusion proteins, regardless of the phosphodomain sequence, was possibly due to their misfolding, which may have masked the host protein binding sites when they were expressed in *E. coli*. This result further suggests that electrostatic interaction alone is unlikely to be sufficient to mediate the CTD-B23 or I2PP2A interaction. Similarly, B23 and I2PP2A have been reported to interact with the basic capsid proteins from the West Nile Virus [31] and the Japanese Encephalitis Virus [32] in a way that is independent of their acidic domains. Little is known about the structure of the hepadnavirus core CTD including the linker region. Our results here suggest that the CTD is likely to adopt a specific conformation in infected cells that may not be attained in bacteria. The known effect of DHBc CTD phosphorylation on its conformation [9] also lends support to the notion that CTD-host protein interactions may be modulated by CTD conformation. When expressed in the mammalian cell lysate (RRL), the CTD was most likely folded correctly as demonstrated by the phosphorylation-induced mobility shift of the full-length DHBc and the isolated CTD in RRL as in living cells [9], [15], and the effect of phosphorylation state on B23 and I2PP2A interactions as observed in the cells.

The linker region where B23 and I2PP2A bind is thought to be responsible for the non-specific RNA/DNA binding activity of DHBc, and together with the viral reverse transcriptase, is essential for packaging specifically the viral pgRNA [8], [14], [16], [33], [34]. The enhancement of B23/I2PP2A-CTD interactions by RNase A treatment suggests that RNA and host protein interactions with this same region may be competitive or mutually exclusive. Indeed, B23 interaction with the human immunodeficiency virus-1 (HIV-1) Rev protein is dissociated by the Rev response element (RRE) RNA [35]. As B23 is known to have non-specific nucleic acid binding activity [36], [37], one potential effect of B23 and I2PP2A may be to modulate the CTD nucleic acid binding activity, thus affecting the packaging of non-specific RNAs vs. the specific viral pgRNA.

The DHBc linker region also harbors a nuclear localization signal (NLS), a cluster of basic residues (**Fig. 1D**), and CTD phosphorylation is thought to negatively regulate core protein nuclear localization [10], [18], [20]. Consistent with this, we found that the GST-fusion protein containing the linker region (196–228) alone or the entire the CTD (196–262) with alanine, but not aspartate, substitutions at the phosphorylation sites was localized mainly to the nucleus (Ludgate and Hu, unpublished results). Binding of B23 and/or I2PP2A to the linker region could affect the function of the DHBc NLS and may thus regulate its nuclear import. Furthermore, B23 is a shuttling protein, moving constantly between the nucleus and the cytoplasm [38]. Similarly, both HBc and DHBc are also thought to shuttle between the nucleus and cytoplasm [18], [28]. It is thus conceivable that B23 may be involved in shuttling the core protein between the nucleus and the cytoplasm, a process that also appears to be regulated by the phosphorylation state of the CTD [18], [20], [28]. In this regard, it is interesting to note that B23 interacts with the NLS of the HIV-1 Rev protein and stimulates Rev nuclear import [35], [39]–[41].

Efforts to directly determine the role of B23 and I2PP2A in core functions and in viral replication in vivo have so far been complicated by the multifunctionality of B23 and I2PP2A, which affect multiple cellular functions including transcription, translation, chromatin remodeling, and cell signaling [42]–[46]. Ectopic overexpression or siRNA mediated knockdown of these proteins affected DHBc expression in transfected cells, independent of the CTD phosphorylation state (Ludgate and Hu, unpublished results). These pleiotropic effects rendered the analysis of any effects of B23 or I2PP2A directly on core function or viral DNA replication in vivo, as opposed to indirect effects due to changes in cellular physiology, rather difficult. Development of cell-free systems that can recapitulate core protein functions that are regulated by dynamic CTD phosphorylation, including pgRNA packaging and reverse transcription, will greatly facilitate future efforts to determine the effects of these and other core-interacting host proteins on core protein functions.

## Materials and Methods

### Plasmids and antibodies

pCMV-DHBV/C^-^ expresses a core-defective DHBV genome and pcDNA-Dcore expresses wild type (WT) DHBc [15]. Similarly, pcDNA-Dcore-SSAAAA, -SSDDDD, -AAAAAA, and -DDDDDD express DHBc with S/T-to-alanine (A) or -aspartic acid (D) substitutions at the four downstream phosphorylation sites (T239, S245, S257, S259), or at all six phosphorylation sites (S230 and S232 in addition to the aforementioned four sites) [15]. pCMV-δXM was derived from pCMV-DHBV by deleting DHBV sequences from 1212 to 2370 and allows expression of only the core protein [24]. As above, the phosphosite mutants δXM-SSAAAA, -SSDDDD, -AAAAAA, and -DDDDDD express DHBc protein with S/T-to-A or -D substitutions at the four downstream phosphorylation sites or at all six phosphorylation sites in the δXM background. DHBc CTD (DCC) coding sequences (from 196 or 229 to 262 or from 196–228, WT and phosphorylation site mutants) were subcloned from pcDNA-Dcore plasmids to pGEX-KT [47] or pEBG [24], downstream of the GST coding sequences, for expression of the GST-DHBc CTD (GST-DCC) fusion proteins in bacteria and mammalian cells, respectively. For in vitro expression of the GST-DCC fusion proteins, the coding sequences were subcloned into the pCI vector (Promega) downstream of the T7 promoter. pSP64pA-Dcore contains the WT DHBc coding sequence downstream of the SP6 promoter in the pSP64pA vector (Promega). I2PP2A-Flag-HA expresses the human I2PP2A protein with C-terminal Flag and HA tags in the pcDNA3.1 vector [48]. p3X-Flag-CMV-14-NPM (referred to as 3X-Flag-B23) expresses human B23/nucleophosmin protein with C-terminal 3X-Flag tags [49].

HBc CTD (HCC) coding sequences from 141 to 183 (WT and phosphorylation site mutants S-to-A or S-to-glutamic acid (E) at the three major SP sites: S155, S162, and S170) were generated by PCR amplification and subcloned to pEBG [24], downstream of the GST coding sequences, for expression of the GST-HBc CTD (GST-HCC) fusion proteins in mammalian cells.

A rabbit polyclonal antibody against DHBc was generously provided by Dr. William Mason [50]. Monoclonal mouse anti-Flag M2 (Sigma), mouse anti-HA.11 (Covance), mouse anti-GST (Thermo Scientific), mouse anti-NPM (ARP), polyclonal rabbit anti-B23 (C-19) and rabbit anti-I2PP2A (H-120) (Santa Cruz) were commercially obtained. Goat anti-rabbit IgG HRP-conjugated antibody (Southern Biotech) and goat anti-mouse HRP-conjugated antibody (Invitrogen) were used as secondary antibodies.

### Cell line maintenance and transient transfection

HEK293T (human embryonic kidney) cells were obtained from Dr. Zhijun Luo (Boston Medical Center) [24], [25] and were routinely cultured in Dulbecco's Modified Eagle's Medium (DMEM)/F12 supplemented with 10% fetal bovine serum (FBS; Hyclone), as previously described [15]. HEK293T cells were transfected by calcium phosphate precipitation as described previously [15]. Protein expression was normally analyzed on the third day post-transfection and viral DNA replication on the fifth day post-transfection.

### GST-fusion protein expression and purification

GST-fusion proteins were purified from bacteria as previously described [51] with the exception that BL21 (DE3) CodonPlus cells were used in this study. GST-fusion proteins were purified from HEK293T cells as described previously [24] with minor modifications. Briefly, HEK293T cells were transfected with GST-fusion protein expression plasmids. On the third day post-transfection, cells were lysed in 1 ml Lysis Buffer containing 50 mM Tris, pH 8.0, 150 mM NaCl, 1 mM EDTA, 1% NP-40, and protease inhibitors (complete protease inhibitor cocktail, Roche) and phosphatase inhibitors (10 mM sodium fluoride, 50 mM β-glycerophosphate, 10 mM sodium pyrophosphate, and 2 mM sodium orthovanadate). Cell debris was removed after centrifugation of the lysate and the cleared supernatant was treated with RNase A (67 µg/ml; Sigma) for 1 hour at room temperature with gentle agitation. The supernatants were removed from the precipitates and used for purification using glutathione (GSH) affinity resin.

For ^35^S-metabolic labeling, three days post-transfection, 60 mm dishes of HEK293T cells were washed twice with 4 ml cysteine/methionine-free DMEM (Invitrogen). Cells were incubated in 0.5 ml cysteine/methionine-free DMEM supplemented with 10% dialyzed FBS (HyClone) for 1 hour to deplete cellular stores of cysteine and methionine. One mCi [^35^S]cysteine/methionine Express Protein Labeling Mix (>1000 Ci/mmol; Perkin Elmer) was added to the cells which were then incubated for an additional 6 hours. GST-tagged proteins were purified as described above.

The [^32^P]orthophosphate metabolic labeling procedure was carried out as described [9]. Briefly, two days post-transfection, HEK293T cells were washed twice with 4 ml phosphate-free DMEM (Invitrogen). Cells were phosphate-starved for 6 hours in 4 ml phosphate-free DMEM supplemented with 10% dialyzed FBS, then incubated for an additional 16 hours at 37°C with 100 µCi [^32^P]orthophosphate (9,000 Ci/mmol; 150 mCi/ml; Perkin Elmer). GST-tagged proteins were purified as described above.

### GST-pulldown using fusion proteins expressed in bacteria

GST-DCC fusion proteins were purified from bacteria as described earlier, except that the proteins were left bound to the GSH resin without elution. HEK293T cells were lysed in 1 ml Lysis Buffer and treated with RNase A (67 µg/ml) for 1 hour at room temperature with gentle agitation. The supernatants were removed from the precipitates and were applied to the fusion protein-bound GSH resin for 4 hours at 4°C, rotating. The resin was collected by 2 minute centrifugation at 800 x *g* and unbound proteins were removed. The resin was washed extensively in Lysis Buffer containing 1 mM phenylmethylsulfonyl fluoride (PMSF; Roche) and 10 mM dithiothreitol (DTT; American Bioanalytical). Bound proteins were eluted in 20 mM GSH (Sigma) in 200 mM Tris, pH 8.0 with the protease and phosphatase inhibitors described above and analyzed by SDS-PAGE and detected by protein staining and western blotting.

### Preparation of samples for mass spectrometric analysis

GST-DCC fusion proteins purified from HEK293T cells were resolved by SDS-PAGE and visualized by Coomassie Brilliant Blue (CB) staining. Co-purifying protein bands of interest were digested in-gel with trypsin and prepared for MS analysis as previously described [11].

### Coimmunoprecipitation

HEK293T cells were cotransfected with I2PP2A-Flag-HA or 3X-Flag-B23 and GST-DCC fusion construct (1∶1 ratio). On the third day post-transfection, cells were lysed in Lysis Buffer. Lysates were digested with RNase A as described earlier. The digested lysates were clarified and applied to protein A/G resin (Pierce) that had been prebound to antibody, either anti-Flag (Sigma) alone (for 3X-Flag-B23 IP), a mixture of anti-Flag and anti-HA (Covance) (for I2PP2A-Flag-HA IP), or a non-specific mouse IgG antibody. The samples were incubated for 4 hours at 4°C, rotating end-over-end. The beads were collected by centrifugation and washed extensively as described earlier. Bound proteins were eluted by boiling in SDS sample buffer and resolved by SDS-PAGE. Bound proteins were assessed by western blotting [52]. GST-DCC fusion proteins were detected with the antibody against GST and I2PP2A or B23 expression was detected with antibody against I2PP2A or B23, respectively.

### In vitro translation and coimmunoprecipitation

Full-length DHBc or GST-DCC fusion proteins were expressed and [^35^S]methionine-labeled using the TnT in vitro rabbit reticulocyte lysate (RRL) transcription and translation coupled kit according to manufacturer's recommendations (Promega). Where indicated, translation products were treated with 1 U calf intestinal alkaline phosphatase (CIAP; New England BioLabs) per µl translation for 16 hours at 37°C. DNase I (100 µg/ml; Roche) and RNase A (100 µg/ml) were then added to the reactions, after which the translation reactions were incubated at room temperature for 1 hour with gentle agitation. Precipitates were removed and each reaction was evenly divided into three aliquots for binding to the I2PP2A-Flag-HA resin, 3X-Flag-B23 resin or untransfected control resin, as prepared above from HEK293T cells. Unbound proteins were removed after collection of the resin by centrifugation. Beads were washed extensively in Lysis Buffer containing 0.1% NP-40. Bound proteins were eluted by boiling in SDS sample buffer and resolved by SDS-PAGE. The gels were stained with Imperial Protein Stain (Pierce) to visualize antibody chains and to ensure samples had been loaded equally. ^35^S-labeled full-length DHBc or GST-DCC fusion proteins were visualized by autoradiography.

### Analysis of viral protein expression and DNA synthesis

Viral DNA was isolated from transfected cell lysates as previously described [15]. Total protein expression was analyzed by SDS-PAGE and western blotting. Core protein expression was detected using an antibody against DHBc [50]. Viral core DNA was resolved on 1.5% agarose gels. Following transfer to Hybond-N membrane (GE Amersham), viral DNA was detected by Southern blotting with a DHBV DNA probe.
